# A 2-Week Course of Enteral Treatment with a Very Low-Calorie Protein-Based Formula for the Management of Severe Obesity

**DOI:** 10.1155/2015/723735

**Published:** 2015-05-06

**Authors:** Giuseppe Castaldo, Luigi Monaco, Laura Castaldo, Paolo Sorrentino

**Affiliations:** ^1^Clinical Nutrition Unit, A.O.R.N. “San Giuseppe Moscati”, 83100 Avellino, Italy; ^2^Ultrasonography Unit, A.O.R.N. “San Giuseppe Moscati”, 83100 Avellino, Italy; ^3^Liver Unit, A.O.R.N. “San Giuseppe Moscati”, 83100 Avellino, Italy

## Abstract

*Background*. Multiple weight loss failures among obese patients suggest the design of new therapeutic strategies. We investigated the role of 2-week course of enteral treatment with a very low-calorie protein-based formula in the management of severe obesity.* Methods*. We evaluated the feasibility, safety, and efficacy of 2-week continuous administration of a protein-based formula (1.2 g/kg of ideal body weight/day) by nasogastric tube in severely obese adults (body mass index (BMI) ≥ 40 kg/m^2^).* Results*. In total, 364 patients (59% women; BMI = 46.6 ± 7.2 kg/m^2^) were recruited. The intervention was discontinued within 48 hours in 26 patients, due to nasogastric tube intolerance. No serious adverse events occurred. During the first and the second week, 65% and 80% patients, respectively, reported no side effects. All biochemical safety parameters were affected by the intervention, particularly uric acid (+45%) and aminotransferases (+48%). In the other cases the change was negligible. We observed significant weight loss (5.7 ± 2.3%) and improvement in blood pressure and glucose and lipid metabolism parameters (*P* < 0.001).* Conclusions*. A 2-week course of enteral treatment with a very low-calorie protein-based formula appeared a feasible, likely safe, and efficacious therapeutic option to be considered for inclusion into a composite weight loss program for the management of severe obesity. This trial is registered with ClinicalTrials.gov Identifier: NCT01965990.

## 1. Introduction

Excess body weight, from overweight to overt obesity, is associated with adverse health outcomes [1]. In view of the time-trends of the obesity epidemic [2] and the related cost burden [3], the search for effective strategies for weight reduction and long-term maintenance of weight loss (WL) is at the top of the agenda of public health systems.

The current first-line strategy includes several treatment options and dietary interventions to be implemented together with an exercise program [4]. Unfortunately, compliance with intervention in the long-term is difficult. It is not infrequent to observe people following one diet after another and experiencing multiple failures which, in turn, lead to higher body weight and adverse consequences on body composition and fat distribution [5]. The higher the number of attempts, the more difficult the adherence to further interventions. The use of antiobesity drugs could be considered, but many of them have recently been banned [6]. In the presence of severe (body mass index >40 kg/m^2^) or complicated obesity, bariatric surgery could be proposed. This therapeutic option is effective [7] but is not devoid of complications and may be irreversible [8]. Obesity-related complications, such as diabetes, hypertension, or sleep apnea, are likely to occur more frequently with increasing body mass index (BMI) and rapid and considerable WL is mandatory to curtail such risks.

In this scenario, alternative treatment options are warranted. About 40 years ago, after the introduction of protein-sparing modified fast (PSMF) achieved through the use of oral high-protein foods or liquid formula diets by Blackburn and Bistrian, several studies evaluated its effectiveness and safety [9, 10]. They showed that responsible and supervised very low-calorie diets (VLCDs) could be considered safe and appropriate therapy for obesity [11].

The purpose of the present study was to investigate the potential role of a 2-week course of enteral treatment with a very low-calorie protein-based formula in the management of severe obesity.

The rationale of this treatment option rests on the following considerations: (1) VLCDs appear to be able to reduce cardiovascular risk rapidly and effectively [9, 10, 12]; (2) VLCDs induce considerable short- and long-term WL [11]; (3) optimal compliance with the intervention, as active participation of the patient, is not required; (4) continuous administration of the intervention formula by enteral route enables the maintenance of the body amino acid pool.

Before being proposed for clinical use, a new WL program should be scientifically evaluated [13, 14]. Accordingly, in the present study, attention was focused not only on efficacy in terms of improving the cardiometabolic risk profile but also on the feasibility and safety of the procedure.

## 2. Materials and Methods

### 2.1. Design

This is an open-label interventional study in severely obese, adult out-patients.

### 2.2. Subjects

All subjects consecutively (from April 2010 to February 2013) attending the Clinical Nutrition Unit of our institution for a WL program were screened for inclusion into the study. Patients had to fulfill the following criteria to be eligible: age ≥ 18 years, severe obesity (body mass index (BMI) ≥ 40 kg/m^2^) [4], and history of multiple failures in WL programs. Exclusion criteria were age ≥ 70 years, type-2 diabetes mellitus on insulin, a psychiatric disorder, previous (<1 year since last chemo- or radiotherapy) or current neoplastic disease, established vascular disease, recent history of diet-induced or unintentional WL (within the previous 6 months), moderate-to-severe heart failure, arrhythmia, renal failure (creatinine > 1.5 mg/dL), current hepatitis, liver cirrhosis, any type of gastrointestinal disease, moderate-severe hypoalbuminemia (<3.0 g/dL), altered serum electrolytes, established gouty and any other contraindication to enteral nutrition, and refusal to give written informed consent.

### 2.3. Intervention

Patients were prescribed a homemade very low-calorie (~6 kcal/kg of ideal body weight/day) protein-based formula (2000 mL per day) by enteral route for 14 days. A polyurethane 8-French nasogastric tube was inserted on day 1 and removed at the end (day 15) of the cycle. The nutritional formula was administered continuously (24 hours a day) by means of a small, light, and rechargeable peristaltic feeding pump (Flocare Infinity, Nutricia, Italy) equipped with phthalates and latex-free infusion line. All patients received the enteral nutrition bags at home every day. The enteral nutrition bag and the pump were supplied in a backpack, thus enabling patients to continue to lead a normal life, pursuing their activities of daily living as usual.

The intervention formula was made up of a fixed amount of amino acids (arginine, ornithine-alpha-ketoglutarate, taurine, cysteine, tryptophan, hydroxyproline, and citrulline) and a variable quantity of high-quality (milk whey) proteins (Nepisond; Gefaldiet Service srl, Italy) in order to reach a total protein content of 1.2 g per kilogram of ideal body weight [9] calculated by Lorentz's equations: Height − 100 − (Height − 150)/4, for men; Height − 100 − (Height − 150)/2.5, for women. Other components of the formula were prolipolytic substances (coenzyme Q10 and L-carnitine), linseed oil (10 grams per day), policosanol, fructooligosaccharides, potassium, sodium, and magnesium chloride. Details of the composition of the formula are provided in Table 1. The enteral nutritional treatment was also complemented by the daily oral administration of a complete (100% of recommended dietary allowances) multivitamin-multimineral supplement, alkalizing substances (calcium carbonate, 1500 mg daily; magnesium carbonate, 850 mg daily; potassium bicarbonate, 500 mg daily; sodium bicarbonate, 1500 mg daily; potassium citrate, 500 mg daily), and herbal remedies commonly marketed for their diuretic, antioxidant, antidiabetic, anti-inflammatory, hepatoprotective, and detoxifying properties (equisetum, nettle, hawthorn, silymarin,* Orthosiphon*, and fucoxanthin) [15–20]. All patients were also prescribed treatment with a proton pump inhibitor and ursodeoxycholic acid: 900 mg daily and 450 mg daily for those with and without documented (by ultrasonography) cholestatic liver disease, respectively.

Patients were allowed to drink water or unsweetened drinks freely (not tea or coffee) during the day. A minimum intake of 2 liters daily was recommended. In patients with a history of kidney stones, the amount was increased to 3 liters.

Before the course, all treatments with hypoglycemic agents and diuretics were discontinued to avoid unintended hypoglycaemia and electrolyte imbalance. Other treatments with anti-hypertensive medications and uric acid and lipid-lowering drugs were left unchanged. Finally, a bowel preparation protocol was adopted for dinner two days before the start of intervention: on day 1, 500 mL of probiotic fermented skimmed milk + vegetable side dish seasoned with olive oil + herbal laxative syrup; on day 2, 500 mL of probiotic fermented skimmed milk + vegetable side dish seasoned with olive oil. During the intervention, no use of laxatives was allowed in order to avoid potassium and bicarbonate loss.

### 2.4. Assessments

#### 2.4.1. Anthropometry

All the subjects had height (to the nearest 0.5 cm) and body weight (to the nearest 0.1 Kg) measured by the same calibrated flat scales equipped with a telescopic, vertical steel stadiometer according to standard procedures. The BMI was derived accordingly (weight [kg] and height [m] squared; kg/m^2^). Waist and hip circumferences (WC and HC, resp.) were assessed (to the nearest 0.5 cm) using a plastic flexible tape. Placing the tape perpendicular to the long axis of the body and parallel to the floor, WC and HC were measured at the midpoint between the lowest rib and the iliac crest and around the largest portion of the buttocks, respectively. The waist-hip ratio (WHR) was also calculated [4].

#### 2.4.2. Hematology and Biochemistry

Venous blood samples were drawn after 8 to 12 hours of fasting and the following parameters were assessed on the same day by our institutional laboratory using conventional automated analyzers and commercial kits: hemoglobin, total lymphocyte count, blood urea nitrogen (BUN), creatinine, uric acid, glucose, insulin, C-peptide, glycated hemoglobin, growth hormone (GH), insulin-like growth factor 1 (IGF-1), total cholesterol, high-density and low-density lipoprotein cholesterol (HDL and LDL, resp.), triglycerides, apolipoproteins A-I and B (ApoA-I and ApoB, resp.), albumin, serum enzymes (cholinesterase, aspartate aminotransferase (AST), alanine aminotransferase (ALT), gamma glutamyl transferase (*γ*-GT), creatine phosphokinase (CPK), and lactate dehydrogenase (LDH)), and electrolytes (sodium, potassium, magnesium, calcium, and phosphorus). Insulin resistance was estimated by the homeostasis model assessment of insulin resistance (HOMA-IR) [21]. The triglyceride/HDL cholesterol ratio and ApoB/ApoA-I ratio were also considered [22, 23].

#### 2.4.3. Blood Pressure

Systolic and diastolic blood pressure (SBP and DBP, resp.) were measured by appropriately sized standard sphygmomanometers after having the patient seated for at least 5 minutes in a chair, with feet on the floor and arm supported at heart level. The average of three measurements, obtained at 2-minute intervals, was used for the analysis [24]. Heart rate was also recorded.

#### 2.4.4. Bile Duct Ultrasonography

Patients were also screened for the presence of cholestatic liver disease (overt cholelithiasis or biliary sludge).

Data on these parameters were collected at baseline (day 0; tube placement) and at the end (day 15; tube removal) of the intervention, before any pharmacological treatment was reintroduced. Patients were also asked to check their ketosis status daily by means of urine sample spot checks.

### 2.5. Study Outcomes

#### 2.5.1. Feasibility

It was defined by the necessity to discontinue the intervention. The causes of the discontinuation were to be recorded.

#### 2.5.2. Safety

Patients were asked to report the onset of any of the following side effects daily, using a self-administered questionnaire: asthenia, headache, dizziness, fainting, orthostatic hypotension, heartburn, nausea, vomiting, palpitations, muscle cramps, hunger, and constipation. Safety was also assessed by the evaluation of changes in the following hematological and biochemical parameters and, for those presenting with normal values, by the excursion outside of the reference ranges of our laboratory (new cases): hemoglobin, total lymphocyte count, BUN, creatinine, uric acid, albumin, serum enzymes (cholinesterase, AST, ALT, *γ*-GT, CPK, and LDH), and electrolytes (sodium, potassium, magnesium, calcium, and phosphorus).

#### 2.5.3. Efficacy

It was defined by the changes (increase (HDL, ApoA-I, and GH) or more frequently the reduction (all the others)) in the following study parameters: body weight, BMI, WC, HC, WHR, uric acid, glucose, insulin, HOMA-IR, C-peptide, glycated hemoglobin, IGF-1, total cholesterol, HDL, LDL, triglycerides, triglyceride/HDL ratio, ApoB, ApoB/ApoA-I ratio, AST, ALT, and *γ*-GT.

### 2.6. Statistical Analysis

All statistical analyses were performed using the software MEDCALC for Windows, Version 11.3.0.0 (MedCalc Software, Mariakerke, Belgium). The level of significance was set at the two-tailed *P* value <0.05.

Safety parameters were analyzed in and reported for the per-protocol (PP) population. However, the analysis of efficacy parameters was conducted according to the intention-to-treat (ITT) principle. Accordingly, all patients who had been assessed at baseline were included in the ITT population. The value observed at baseline was used in the analyses for dropouts.

Data were presented as mean and standard deviation (SD) or counts and percentage, as appropriate.

Group comparisons were performed using Fisher's exact test (categorical variables) and Student's *t*-test or Wilcoxon's test (continuous variables).

Changes in study parameters were investigated by Student's *t*-test or Wilcoxon's test for paired data, while proportions were compared with Fisher's exact test. A set of secondary analyses was also performed by building generalized linear regression models, including changes (difference = final − baseline) in study parameters as alternative dependent variables and age, gender, and diabetes as independent variables. Finally, logistic regression analysis of baseline features was considered to investigate the alterations (values below or above the lower and the upper limit of laboratory range, resp.) in safety parameters occurring in at least 10% of patients.

## 3. Results

In total, 364 patients (59% females; BMI (mean ± SD), 46.6 ± 7.2 kg/m^2^) were recruited. Based on ideal body weight (mean ± SD, 61.2 ± 7.1 kg) the mean (±SD) daily protein and calorie intake were 73.4 ± 8.6 g/day and 383 ± 34 kcal/day, respectively.

### 3.1. Feasibility

Overall, the intervention was well tolerated. Twenty-six patients (7%; 13 men and 13 women) had to discontinue the intervention within 48 hours. Discontinuation was only due to nasogastric tube intolerance (physical discomfort or social reasons). The features of dropouts were similar to those of completers (Supplementary Table 1, available online at http://dx.doi.org/10.1155/2015/723735). Finally, compliance was optimal in completers, as confirmed by the evaluation of urinary ketosis.

### 3.2. Safety

No serious adverse events occurred during the intervention. Throughout the intervention period, constipation was the most frequently reported side effect (8.6% and 10.9% during the first and the second week, resp.). Other frequent (>10%) side effects reported during the first week were headache and hunger. However, their frequency was significantly reduced during the second week of the study. The same applied to other symptoms, with the exception of muscle cramps. Nonetheless, prevalence was almost below 5%. The frequency of self-reported side effects throughout the study is reported in detail in Table 2. Overall, “no side effects” during the first and the second week was reported by 220 (65%) and 270 (80%) patients, respectively.

In respect to hematological and biochemical variables (Table 3), looking at mean values in the whole population, all safety parameters were affected by the intervention. An increase in the following values was observed: hemoglobin, creatinine, uric acid, albumin, AST, ALT, CPK, LDH, potassium, calcium, and phosphorus. However, also a reduction in the following was detected: total lymphocyte count, BUN, cholinesterase, *γ*-GT, sodium, and magnesium. The greatest changes were observed for uric acid (+45%), AST (+48%), and ALT (+47%) levels, although only a limited number of patients reported values slightly higher than two times the upper limit of normality (uric acid, *n* = 3; AST, *n* = 13; ALT, *n* = 34). In the other cases the change was almost negligible, as most patients retained values within the normal ranges for our laboratory (as suggested also by 95% confidence intervals of the mean difference). Interestingly, according to regression analysis, the most frequent (frequency > 10%) alterations (increase above the upper limit of laboratory range) in biochemical parameters (uric acid, ALT, AST, and CPK) occurred in male subjects (*P* < 0.01 for all). Changes in uric acid were also more likely to occur in younger patients (*P* < 0.01). Finally, the increase in ALT was higher in those who had normal values at baseline (for ALT: +75% versus +37%, *P* < 0.001).

### 3.3. Efficacy

Despite similar age (women, 40.1 [SD, 10.8] versus men, 41.1 [SD, 10.3]; *P* = 0.422) and degree of obesity, as expected, male patients were characterized by more evident abdominal adiposity and abnormalities in cardiometabolic risk profile (blood pressure and both glucose and lipid metabolism parameters; Supplementary Table 2). However, the prevalence of diabetes was almost comparable between genders (*P* = 0.072): 18.6% and 26.8%, in women and men, respectively.

The intervention resulted in significant WL (ITT population, 5.7% [SD, 2.3]; PP population, 6.1% [SD, 1.8]; Table 4). This applied to both genders (ITT population: men, 5.9% [SD, 2.5] versus women, 5.5% [SD, 2.2]; *P* = 0.118; Supplementary Table 2).

WL resulted in significant improvement in blood pressure, in all anthropometric variables and most hematological and biochemical parameters. As expected, uric acid and ApoB/ApoA-I ratio showed an increase, while HDL, ApoA-I, and IGF-1 were significantly decreased (Table 4). Results were confirmed by analyses in PP population. These findings were substantially confirmed by gender-specific analyses (Supplementary Table 2).

In secondary efficacy analyses (multivariable linear regression; Table 5), it was observed that the improvement in glucose control-related variables (glucose, insulin, HOMA-IR, C-peptide, glycated hemoglobin, triglycerides, and triglyceride/HDL ratio) and blood pressure (both systolic and diastolic) was even greater in diabetic patients.

In respect to the improvement of cardiometabolic profile, we report also that the diuretic therapy was no longer deemed necessary while oral hypoglycemic agents were reintroduced at half of the initial dose.

## 4. Discussion

The present study has shown that a 2-week course of PSMF, achieved through the administration of a very low-calorie protein-based formula by enteral route, is a feasible, safe, and effective procedure to be taken into due consideration for a composite weight loss program for the management of severe obesity.

The effectiveness of VLCD has been reported to rest on its appetite-suppressing ketogenic nature, which mimics the anorexia of starvation, and the satiating effect associated with its high-protein content [9, 10]. However, compared to previous studies in which the dietary regimen was achieved by pulsatile administration of low-calorie, low-carbohydrate, high-protein foods or liquid formula, the use of the enteral nutrition technique enabled around-the-clock administration. The main advantage of this approach is the possibility of keeping the patient in steady-state conditions, reducing the difficulties in complying with a formula-based therapeutic regimen mainly due to hunger or palatability/acceptability as much as possible [25]. In a state of complete absence of carbohydrates, which can be achieved only with the use of a specifically designed liquid formula, ketogenesis and enteral stimulation are constant. It could not be excluded that compliance with and acceptability of the intervention were also to be ascribed to the fact that a feeding tube is perceived as real medical therapy. Moreover, the presence of the tube may emphasize the social disruptiveness previously reported for VLCDs [25]. However, this approach can reasonably be proposed as a short-term intervention (2–4 weeks). Compared to the other VLC interventions [11] that commonly provide about 1000 kcal/day for up to 12 weeks, a PSMF approach provides about 400 kcal/day. Nonetheless, protein and micronutrients malnutrition is avoided with adequate protein administration and micronutrients supplementation. This is a relevant issue, as most prolonged low-calorie dietary interventions shift to intake associated with risk of micronutrients inadequacy [26].

In our study, discontinuation of therapy was unrelated to adverse effects and was driven only by intolerance to the tube, either for physical or social reasons. Self-reported side effects were few and mainly limited to the first days of the intervention. Previous studies have reported the occurrence or intensification of emotional disturbances, such as depression and anxiety during intensive WL [27, 28]. Patients with psychiatric disorders were excluded during the baseline assessments and these symptoms were not observed in our experience. As, in order to minimize adverse effects, it is not recommended to prescribe dietary regimens with calorie content lower than basal metabolic rate, we also investigated the safety of this experimental intervention. With exception of serum uric acid and aminotransferases, most changes in biochemical safety parameters still fell within the normal ranges. Ketogenic regimens are known to increase uric acid. This appears to generally occur during the first 6 weeks, with normalization during maintenance of a lower weight. No attack of acute gouty arthritis was observed, but an incidence of up to 1 percent of this disorder has been reported during longer VLCD programs [10]. The increase in aminotransferases is also consistent with the intense lipolytic activity and the intense flow of fatty acids to the liver [29]. Intense lipolysis is likely reflected also by restored growth hormone incretion after WL [30]. However, contrary to previous evidence, WL was associated with a reduction in circulating IGF-1, which is consistent with short-term starvation and the similar effect on the immune system [30, 31], as reflected by the subclinical reduction in lymphocyte count. It is reasonable to argue that most of these changes are transient, without relevant clinical implications, and can be normalized by returning to a balanced oral diet. Moreover, they were of limited entity and the consequence of an intervention of limited duration. Accordingly, the appropriate selection of the candidates performed is an important step.

The intervention investigated herein has also proved to be effective in reducing body weight and improving the cardiometabolic risk profile. This was particularly evident in diabetic patients, thus supporting the efficacy of VLCDs in rapidly modifying cardiovascular risk [9, 12]. However, it also appeared to induce a detrimental reduction in HDL and ApoA-I levels and an increase in ApoB/ApoA-I ratio, despite the significant decrease also in ApoB, which is secondary to the restriction of saturated fat and cholesterol [32].

Current guidelines [4] recommend, as initial goal, a WL of 10% from baseline, to be achieved approximately over 6 months. In the present experience of two-week PSMF by enteral route we observed a mean WL of ~6%. We could hypothesize that the repetition of 2 or 3 courses of this therapy could bring consistent benefits to the patient. However, despite optimal compliance and acceptability during enteral nutrition, the intervention lasts only 14 days and the patient still requires constant monitoring and follow-up evaluations. The present treatment procedure, alternated with periods of closely monitored oral diet, was designed to be included in a composite dietary rehabilitation program, with the gradual introduction of a balanced Mediterranean-like diet. In respect to this, an apparent advantage may be the transient postdiet hypophagia observed after switching from a ketogenic diet to ad libitum food [33]. Nonetheless, thinking about the overall management pathway of severe obesity, a potential preoperative application to reduce anesthetic risk could be hypothesized.

PSMF and more extensively prolonged VLCDs have raised concerns and criticisms because of the risk of complications, which may occur not only during the intervention but also during the “refeeding” phase and are mainly related to electrolyte disturbances. These include some major problems, such as nephrolithiasis or arrhythmias, and other minor issues (headache, nausea, occasional vomiting, bad breath, fatigue, muscle cramps, and constipation) [34]. However, our study shows that most complications can be easily prevented with the use of high-quality proteins and relevant electrolyte, vitamin, and mineral supplements [10, 34]. Indeed, responsible use of this therapeutic regimen for short-term periods and careful selection of the candidates are mandatory. In the first instance, our treatment protocol was investigated in severely obese patients with a history of multiple failures. Accordingly, it is not proposed as first-line therapy, but as an alternative therapy before more advanced approaches, such as surgery, in high-risk patients with serious difficulties in complying with further dietary regimens.

Finally, another concern with VLCDs is weight regain. On the one hand, initial WL appears to predict lower body weight at follow-up. On the other, rapid WL has been also associated with greater regain during the maintenance phase [11]. Indeed, the therapy does not engage the patient in life style and food choice changes necessary to be successful in long-term weight reduction. The long-term effects of the present treatment protocol, after inclusion into a composite weight management program, clearly need to be investigated and data collection is ongoing. Studies are also needed to measure changes in nutritional status, body composition, and energy expenditure, although evidence supports the beneficial effect of low-carbohydrate, high-protein diets and the use of milk whey proteins [35, 36].

The strengths of our study are the large study population, the rigorous selection of the patients, and the standardized and responsible approach.

However, also its limitations should be taken into account. First, this was an observational study and randomized trials including control groups—treated either with standard hypocaloric diet or oral VLCD—should be performed to achieve a more rigorous evaluation among the plethora of available WL interventions. Second, the present report focused only on data addressing a single enteral course of treatment; the impact of this intervention should be clearly evaluated within the broader context of a long-term composite weight management program. Particularly, in order to further support its use in clinical practice, postintervention normalization of safety parameters needs to be addressed. Finally, the evaluation of self-perceived outcome measures (e.g., hunger or wellbeing) by means of visual analogue scales would have been also informative.

In conclusion, a 14-day course of enteral treatment with a very low-calorie protein-based formula appears to be a feasible, likely safe, and efficacious therapeutic option to be taken into consideration for a composite weight loss program for the management of severe obesity.

## Supplementary Material

Baseline features of the population by intervention status.
For Supplementary Table 2: "Changes in anthropometric, clinical and metabolic features after the intervention and according to gender."

## Figures and Tables

**Table 1:** Composition of the intervention formula provided daily by enteral route.

Component	Amount
Total volume, mL	2000
Total protein content^∗^, g/kg of IBW	1.2
Milk whey proteins (as necessary)	
Arginine, g	2.25
Ornithine-alpha-ketoglutarate, g	2.25
Taurine, g	0.45
Cysteine, g	0.45
Tryptophan, g	0.75
Hydroxyproline, g	0.45
Citrulline, g	0.45
Lipids (linseed oil), g	10
Alpha-linolenic acid, g	5.5
L-Carnitine, mg	300
Coenzyme Q10, mg	30
Policosanols, mg	500
Fructooligosaccharides, g	15
Sodium, mg	500
Potassium, mg	3000
Chlorum, mg	3000

IBW: ideal body weight.

^**∗**^Including amino acids.

**Table 2:** Self-reported side effects throughout the study (per-protocol population; *N* = 338).

Side effect	Days 1–7	Days 8–14
	*N* (%)	*N* (%)
Asthenia	17 (5.1)	6 (1.8)
Headache	34 (10.0)	3 (0.9)
Dizziness	2 (0.6)	1 (0.3)
Fainting	4 (1.2)	1 (0.3)
Orthostatic hypotension	25 (7.4)	12 (3.6)
Heartburn	23 (6.8)	11 (3.3)
Nausea	13 (3.8)	3 (0.9)
Vomiting	6 (1.8)	2 (0.6)
Palpitations	4 (1.2)	2 (0.6)
Muscle cramps	7 (2.1)	13 (3.8)
Hunger	34 (10.1)	12 (3.6)
Constipation	29 (8.6)	37 (10.9)

**Table 3:** Changes in hematological and biochemical safety parameters after the intervention (per-protocol population; *N* = 338).

Variable	Baseline	Day 14	Mean difference	*P* value^a^	Laboratory range	New cases	
	[Mean (SD)]	[Mean (SD)]	[95% CI]			Below the lower limit *N* (%)^a^	Above the upper limit *N* (%)
Hemoglobin, g/L	14.0 (1.5)	14.1 (1.4)	0.1 [0.03, 0.19]	0.005	12.0–15.5 (13.5–17.0)	12 (3.6)	5 (1.5)
Lymphocytes, *n*/mm^3^	2334 (651)	1946 (587)	−388 [−446, −330]	<0.001	1300–3600	23 (6.8)	2 (0.6)
Blood urea nitrogen, mg/dL	31 (8)	26.7 (7.5)	−3.9 [−4.8, −3.0]	<0.001	10–50	0	1 (0.3)
Creatinine, mg/dL	0.71 (0.15)	0.77 (0.16)	0.06 [0.05, 0.08]	<0.001	0.55–1.2	7 (2.1)	1 (0.3)
Uric acid, mg/dL	5.6 (1.2)	8.1 (2.3)	2.5 [2.2, 2.8]	<0.001	3.5–7.0	0	178 (52.7)
Albumin, g/L	44.4 (0.5)	45.9 (0.5)	1.5 [−1.0, 2.1]	<0.001	35.0–52.0	2 (0.6)	8 (2.4)
Cholinesterase, UI/dL	9839 (1906)	9617 (1893)	−222 [−351, −94]	<0.001	4250–11250	2 (0.6)	21 (6.2)
AST, UI/dL	23 (10)	34 (17)	11 [10, 13]	<0.001	6–39	0	34 (10.1)
ALT, UI/dL	32 (21)	47 (32)	15 [13, 18]	<0.001	6–34	0	90 (26.6)
*γ*-GT, UI/dL	30 (31)	22 (13)	8 [−10, −5]	<0.001	6–42	0	4 (1.2)
CPK, UI/dL	121 (75)	134 (88)	13 [5, 22]	0.003	24–190	0	37 (10.9)
LDH, UI/dL	424 (87)	438 (98)	14 [5, 23]	0.002	125–600	0	21 (6.2)
Sodium, mEq/L	139 (2.2)	138 (2.5)	−1.0 [−1.5, −0.9]	<0.001	135–153	21 (6.2)	0
Potassium, mEq/L	4.4 (0.3)	4.5 (0.3)	0.1 [0.04, 0.14]	<0.001	3.5–5.3	1 (0.3)	3 (0.9)
Magnesium, mg/dL	1.98 (0.17)	1.93 (0.18)	−0.05 [−0.08, −0.04]	<0.001	1.7–2.6	5 (1.5)	0
Calcium, mg/dL	9.3 (0.4)	9.6 (0.4)	0.4 [0.3, 0.4]	<0.001	8.6–10.5	1 (0.3)	4 (1.2)
Phosphorus, mg/dL	3.4 (0.5)	3.7 (0.5)	0.3 [0.3, 0.4]	<0.001	2.7–4.5	3 (0.9)	15 (4.4)

BMI: body mass index; AST: aspartate aminotransferase; ALT: alanine aminotransferase; *γ*-GT: gamma glutamyl transferase; CPK: creatine phosphokinase; LDH: lactate dehydrogenase.

^a^Baseline versus end of study (by Student's *t*-test for paired data or Fisher's exact test).

**Table 4:** Changes in anthropometric, clinical, and metabolic features after the intervention (efficacy analysis; intention-to-treat population [*N* = 364]).

Characteristic	Baseline	Day 14	*P* value^a^
	[Mean (SD)]	[Mean (SD)]	
Body weight, kg	129.0 (24.2)	121.7 (23.5)	<0.001
BMI, kg/m^2^	46.6 (7.2)	43.9 (7.1)	<0.001
Waist circumference, cm	134.5 (16.4)	129.1 (16.1)	<0.001
Hip circumference, cm	139.0 (15.1)	134.9 (14.8)	<0.001
Waist-hip ratio	0.97 (0.09)	0.96 (0.09)	0.068
Uric acid, mg/dL	5.6 (1.2)	7.9 (2.4)	<0.001
Glucose, mg/dL	98 (27)	82 (17)	<0.001
Insulin, *µ*U/mL	23 (19)	12 (12)	<0.001
HOMA-IR	5.8 (6.0)	2.6 (3.3)	<0.001
C-peptide, ng/mL	4.3 (2.2)	2.7 (1.6)	<0.001
HbA1C, %	5.8 (1.0)	5.6 (0.8)	<0.001
Growth hormone, ng/mL	0.62 (1.39)	165 (2.93)	<0.001
IGF-1, ng/mL	146 (75)	124 (76)	<0.001
Total cholesterol, mg/dL	194 (34)	156 (37)	<0.001
HDL cholesterol, mg/dL	47 (12)	36 (10)	<0.001
LDL cholesterol, mg/dL	125 (31)	99 (38)	<0.001
Triglycerides, mg/dL	135 (75)	100 (38)	<0.001
Triglycerides-HDL ratio	3.3 (2.6)	3.1 (1.7)	0.032
ApoA-I, mg/dL	144 (31)	112 (35)	<0.001
ApoB, mg/dL	103 (43)	89 (36)	<0.001
ApoB/ApoA-I ratio	0.75 (0.43)	0.88 (0.56)	<0.001
SBP, mmHg	134 (11)	126 (9)	<0.001
DBP, mmHg	81 (8.5)	75 (6.7)	<0.001
Heart rate, bpm	73 (3.6)	72 (3.3)	<0.001

BMI: body mass index; HOMA-IR: homeostasis model assessment of insulin resistance; HbA1C: glycosylated hemoglobin; IGF-1: insulin-like growth factor 1; HDL: high density lipoprotein; LDL: low density lipoprotein; ApoA-I: apolipoprotein A-I; ApoB: apolipoprotein B; SBP: systolic blood pressure; DBP: diastolic blood pressure.

^a^Baseline versus end of study by Student's *t*-test for paired data.

**Table 5:** Age-adjusted changes^a^ in cardiometabolic and clinical parameters according to gender and diabetes (generalized linear regression analysis in intention-to-treat population [*N* = 364]).

Characteristic	Mean (SD)^a^	Gender (men versus women)		Diabetes (yes versus no)	
		Difference [95% CI]	*P* value	Difference [95% CI]	*P* value
Weight loss, %	−5.7 (2.3)	−0.4 [−1.0, 0.2]	0.109	0.1 [−0.5, 0.7]	0.853
Waist circumference, cm	−5.4 (3.6)	−0.2 [−1.0, 0.6]	0.537	−0.2 [−1.2, 0.8]	0.612
Hip circumference, cm	−4.1 (3.8)	0.1 [−0.7, 0.9]	0.756	−0.7 [−1.7, 0.3]	0.140
Waist-hip ratio	−0.01 (0.02)	0.001 [−0.003, 0.005]	0.657	0.003 [−0.003, 0.009]	0.268
Uric acid, mg/dL	2.3 (2.1)	0.1 [−0.3, 0.5]	0.714	−0.02 [−0.6, 0.06]	0.942
Glucose, mg/dL	−16 (22)	−2 [−6, 2]	0.275	−19 [−25, −13]	<0.001
Insulin, *µ*U/mL	−11 (16)	−5 [−9, −1]	0.009	−5 [−9, −1]	0.016
HOMA-IR	−3.2 (4.9)	−1.5 [−2.5, −0.5]	0.004	−2.9 [−4.1, −1.7]	<0.001
C-peptide, ng/mL	−1.6 (1.9)	−0.4 [−0.8, 0.0]	0.037	−0.6 [−1.0, −0.2]	0.013
HbA1C, %	−0.2 (0.40)	−0.03 [−0.11, 0.05]	0.438	−0.1 [−0.2, 0.0]	0.004
Growth hormone, ng/mL	1.04 (2.32)	−0.1 [−0.7, 0.5]	0.722	−0.2 [−0.8, 0.4]	0.527
IGF-1, ng/mL	−20 (65)	23 [7, 39]	0.005	−13 [−31, 5]	0.174
Total cholesterol, mg/dL	−39 (31)	5 [−1, 11]	0.137	2 [−6, 10]	0.551
HDL cholesterol, mg/dL	−11 (10)	3 [1, 5]	0.002	2 [0, 4]	0.106
LDL cholesterol, mg/dL	−26 (29)	3 [−3, 9]	0.289	7 [−1, 15]	0.068
Triglycerides, mg/dL	−35 (61)	−13 [−25, −1]	0.031	−25 [−41, −9]	0.001
Triglycerides-HDL ratio	−0.2 (2.0)	−0.3 [−0.7, 0.1]	0.183	−0.8 [−1.4, −0.2]	<0.001
ApoA-I, mg/dL	−30 (40)	8 [−2, 18]	0.116	−4 [−16, 8]	0.480
ApoB, mg/dL	−9 (28)	3 [−5, 11]	0.472	1 [−7, 9]	0.826
ApoB/ApoA-I ratio	0.15 (0.58)	−0.1 [−0.3, 0.1]	0.219	0.1 [−0.1, 0.3]	0.238
SBP, mmHg	−7.7 (10.2)	−2.3 [−4.5, −0.1]	0.035	−2.2 [−4.4, 0.0]	0.047
DBP, mmHg	−5.4 (8.3)	−1.9 [−3.7, −0.1]	0.037	−2.3 [−4.5, −0.1]	0.031
Heart rate, bpm	−1 (3.8)	−0.05 [−0.8, 0.7]	0.906	−0.5 [−1.5, 0.5]	0.327

SD: standard deviation; BMI: body mass index; HOMA-IR: homeostasis model assessment of insulin resistance; HbA1C: glycosylated hemoglobin; IGF-1: insulin-like growth factor 1; HDL: high density lipoprotein; LDL: low density lipoprotein; ApoA-I: apolipoprotein A-I; ApoB: apolipoprotein B; SBP: systolic blood pressure; DBP: diastolic blood pressure.

^a^Changes (in percentage) were computed as follows: final – baseline.

## References

[1] Whitlock G., Lewington S., Sherliker P. (2009). Body-mass index and cause-specific mortality in 900 000 adults: collaborative analyses of 57 prospective studies. *The Lancet*.

[2] Finucane M. M., Stevens G. A., Cowan M. J. (2011). National, regional, and global trends in body-mass index since 1980: systematic analysis of health examination surveys and epidemiological studies with 960 country-years and 9.1 million participants. *The Lancet*.

[3] Wang Y. C., McPherson K., Marsh T., Gortmaker S. L., Brown M. (2011). Health and economic burden of the projected obesity trends in the USA and the UK. *The Lancet*.

[4] Expert Panel on the Identification Evaluation and Treatment of Overweight in Adults (1998). Clinical guidelines on the identification, evaluation, and treatment of overweight and obesity in adults: executive summary. *The American Journal of Clinical Nutrition*.

[5] Cereda E., Malavazos A. E., Caccialanza R., Rondanelli M., Fatati G., Barichella M. (2011). Weight cycling is associated with body weight excess and abdominal fat accumulation: a cross-sectional study. *Clinical Nutrition*.

[6] Derosa G., Maffioli P. (2012). Anti-obesity drugs: a review about their effects and their safety. *Expert Opinion on Drug Safety*.

[7] Buchwald H., Avidor Y., Braunwald E. (2004). Bariatric surgery: a systematic review and meta-analysis. *The Journal of the American Medical Association*.

[8] Hamdan K., Somers S., Chand M. (2011). Management of late postoperative complications of bariatric surgery. *British Journal of Surgery*.

[9] Blackburn G. L., Bistrian B. R., Flatt J. P., Howard A. N. (1975). Role of a protein-sparing modified fast in a comprehensive weight reduction program. *Recent Advances in Obesity Research*.

[10] Kawamura I., Chen C. C., Yamazaki K., Miyazawa Y., Isono K. (1992). A clinical study of protein sparing modified fast (PSMF) administered preoperatively to morbidly obese patients: comparison of PSMF with natural food products to originally prepared PSMF. *Obesity Surgery*.

[11] Franz M. J., VanWormer J. J., Crain A. L. (2007). Weight-loss outcomes: a systematic review and meta-analysis of weight-loss clinical trials with a minimum 1-year follow-up. *Journal of the American Dietetic Association*.

[12] Malandrucco I., Pasqualetti P., Giordani I. (2012). Very-low-calorie diet: a quick therapeutic tool to improve *β* cell function in morbidly obese patients with type 2 diabetes. *American Journal of Clinical Nutrition*.

[13] Tsai A. G., Wadden T. A. (2005). Systematic review: an evaluation of major commercial weight loss programs in the United States. *Annals of Internal Medicine*.

[14] Freedhoff Y., Sharma A. M. (2009). ‘Lose 40 pounds in 4 weeks’: regulating commercial weight-loss programs. *CMAJ*.

[15] Carneiro D. M., Freire R. C., Honório T. C. D. D. (2014). Randomized, double-blind clinical trial to assess the acute diuretic effect of *Equisetum arvense* (field horsetail) in healthy volunteers. *Evidence-Based Complementary and Alternative Medicine*.

[16] Ameer O. Z., Salman I. M., Asmawi M. Z., Ibraheem Z. O., Yam M. F. (2012). Orthosiphon stamineus: traditional uses, phytochemistry, pharmacology, and toxicology. *Journal of Medicinal Food*.

[17] Maeda H. (2015). Nutraceutical effects of fucoxanthin for obesity and diabetes therapy: a review. *Journal of Oleo Science*.

[18] Wu J., Peng W., Qin R., Zhou H. (2014). Crataegus pinnatifida: chemical constituents, pharmacology, and potential applications. *Molecules*.

[19] Vargas-Mendoza N., Madrigal-Santillán E., Morales-González A. (2014). Hepatoprotective effect of silymarin. *World Journal of Hepatology*.

[20] Chrubasik J. E., Roufogalis B. D., Wagner H., Chrubasik S. (2007). A comprehensive review on the stinging nettle effect and efficacy profiles. Part II: urticae radix. *Phytomedicine*.

[21] Matthews D. R., Hosker J. P., Rudenski A. S., Naylor B. A., Treacher D. F., Turner R. C. (1985). Homeostasis model assessment: insulin resistance and *β*-cell function from fasting plasma glucose and insulin concentrations in man. *Diabetologia*.

[22] McLaughlin T., Abbasi F., Cheal K., Chu J., Lamendola C., Reaven G. (2003). Use of metabolic markers to identify overweight individuals who are insulin resistant. *Annals of Internal Medicine*.

[23] Walldius G., Jungner I., Aastveit A. H., Holme I., Furberg C. D., Sniderman A. D. (2004). The apoB/apoA-I ratio is better than the cholesterol ratios to estimate the balance between plasma proatherogenic and antiatherogenic lipoproteins and to predict coronary risk. *Clinical Chemistry and Laboratory Medicine*.

[24] Chobanian A. V., Bakris G. L., Black H. R. (2003). Seventh report of the joint national committee on prevention, detection, evaluation, and treatment of high blood pressure. *Hypertension*.

[25] Wadden T. A., Stunkard A. J., Brownell K. D., Day S. C. (1985). A comparison of two very-low-calorie diets: protein-sparing-modified fast versus protein-formula-liquid diet. *American Journal of Clinical Nutrition*.

[26] Gardner C. D., Kim S., Bersamin A. (2010). Micronutrient quality of weight-loss diets that focus on macronutrients: results from the A to Z study. *The American Journal of Clinical Nutrition*.

[27] Halmi K. A., Stunkard A. J., Mason E. E. (1980). Emotional responses to weight reduction by three methods: gastric bypass, jejunoileal bypass, diet. *American Journal of Clinical Nutrition*.

[28] Stunkard A. J., Rush J. (1974). Dieting and depression reexamined. A critical review of reports of untoward responses during weight reduction for obesity. *Annals of Internal Medicine*.

[29] Cusi K. (2012). Role of obesity and lipotoxicity in the development of nonalcoholic steatohepatitis: pathophysiology and clinical implications. *Gastroenterology*.

[30] Douyon L., Schteingart D. E. (2002). Effect of obesity and starvation on thyroid hormone, growth hormone, and cortisol secretion. *Endocrinology and Metabolism Clinics of North America*.

[31] Bistrian B. R., Blackburn G. L., Scrimshaw N. S., Flatt J. P. (1975). Cellular immunity in semistarved states in hospitalized adults. *The American Journal of Clinical Nutrition*.

[32] Vélez-Carrasco W., Lichtenstein A. H., Welty F. K. (1999). Dietary restriction of saturated fat and cholesterol decreases HDL ApoA-I secretion. *Arteriosclerosis, Thrombosis, and Vascular Biology*.

[33] Honors M. A., Davenport B. M., Kinzig K. P. (2009). Effects of consuming a high carbohydrate diet after eight weeks of exposure to a ketogenic diet. *Nutrition and Metabolism*.

[34] Atkinson R. L. (1986). Very low calorie diets: getting sick or remaining healthy on a handful of calories. *Journal of Nutrition*.

[35] Wycherley T. P., Moran L. J., Clifton P. M., Noakes M., Brinkworth G. D. (2012). Effects of energy-restricted high-protein, low-fat compared with standard-protein, low-fat diets: a meta-analysis of randomized controlled trials. *The American Journal of Clinical Nutrition*.

[36] Frestedt J. L., Zenk J. L., Kuskowski M. A., Ward L. S., Bastian E. D. (2008). A whey-protein supplement increases fat loss and spares lean muscle in obese subjects: a randomized human clinical study. *Nutrition and Metabolism*.

