# Supplementary material for: A 2-Week Course of Enteral Treatment with a Very Low-Calorie Protein-Based Formula for the Management of Severe Obesity
Source: Int J Endocrinol. 2015 May 6;2015:723735. doi: 10.1155/2015/723735 (PMC4438151; doi:10.1155/2015/723735)
Supplement: Supplementary file 1 — Baseline features of the population by intervention status. For Supplementary Table 2: "Changes in anthropometric, clinical and metabolic features after the intervention and according to gender." [file 723735.f1.doc]

**Supplementary Table 1.** Baseline features of the population by intervention status.

________________________________________________________________________________________________________________________

**Completed** **Dropout**

**Characteristic ^a^** (N=338) (N=26) **P-value ^b^**

________________________________________________________________________________________________________________________

**Male**, N (%) 136 (40.2) 13 (50.0) 0.408

**Age**, years 40.8 (10.6) 36.9 (9.7) 0.073

**Body weight**, kg 128.6 (23.9) 133.3 (28.9) 0.347

**Body mass index**, kg/m^2^ 46.6 (7.3) 46.3 (5.9) 0.856

**Waist circumference**, cm 134.3 (16.4) 137.2 (16.5) 0.378

**Hip circumference**, cm 139.1 (15.3) 136.8 (12.0) 0.467

**Waist - Hip ratio** 0.97 (0.09) 1.00 (0.08) 0.055

**Hemoglobin**, g/L 14.0 (1.5) 14.3 (1.3) 0.441

**Lymphocytes**, n/mm^3^ 2334 (651) 2188 (718) 0.275

**Blood urea nitrogen**, mg/dL 31 (8) 28 (6) 0.072

**Creatinine**, mg/dL 0.71 (0.15) 0.73 (0.12) 0.422

**Uric acid**, mg/dL 5.6 (1.2) 5.4 (1.4) 0.247

**Albumin**, g/L 44.4 (0.5) 44.2 (0.6) 0.054

**Cholinesterase**, UI/dL 9839 (1906) 9667 (2295) 0.461

**AST**, UI/dL 23 (10) 26 (17) 0.333

**ALT**, UI/dL 32 (21) 38 (26) 0.119

**γ-GT** , UI/dL 30 (31) 34 (27) 0.433

**CPK**, UI/dL 121 (75) 127 (93) 0.730

**LDH**, UI/dL 424 (87) 408 (67) 0.360

**Sodium**, mEq/L 139 (2.2) 139 (1.5) 0.693

**Potassium**, mEq/L 4.4 (0.3) 4.3 (0.3) 0.052

**Magnesium**, mg/dL 1.98 (0.17) 1.98 (0.16) 0.955

**Calcium**, mg/dL 9.3 (0.4) 9.3 (0.4) 0.666

**Phosphorus**, mg/dL 3.4 (0.5) 3.3 (0.5) 0.315

**Glucose**, mg/dL 98 (27) 100 (28) 0.736

**Insulin**, µU/mL 22 (19) 29 (26) 0.225

**HOMA-IR** 5.6 (5.8) 7.9 (8.1) 0.193

**C-peptide**, ng/mL 4.2 (2.2) 5.0 (2.8) 0.203

**HbA1C**, % 5.8 (0.9) 5.8 (0.9) 0.979

**Growth hormone**, ng/mL 0.64 (1.4) 0.62 (1.3) 0.944

**IGF-1**, ng/mL 145 (75) 159 (75) 0.424

**Total cholesterol**, mg/dL 195 (34) 193 (34) 0.750

**HDL cholesterol**, mg/dL 47 (12) 45 (12) 0.588

**LDL cholesterol**, mg/dL 126 (31) 119 (30) 0.275

**Triglycerides**, mg/dL 134 (76) 136 (55) 0.923

**Triglycerides - HDL ratio** 3.3 (2.6) 3.3 (2.0) 0.862

**Apolipoprotein** **A-I**, mg/dL 142 (30) 144 (25) 0.749

**Apolipoprotein** **B**, mg/dL 97 (24) 97 (32) 0.977

**ApoB/ApoA-I ratio** 0.72 (0.39) 0.70 (0.26) 0.593

**SBP**, mmHg 134 (11) 132 (12) 0.398

**DBP**, mmHg 81 (8.5) 79 (9.7) 0.350

**Heart rate**, bpm 73 (3.6) 73 (4.2) 0.652

**Diabetes**, N (%) 73 (21.6) 7 (26.9) 0.622

***Treated***, N (%) 59 (17.5) 5 (19.2) 0.791

**Statins use**, N (%) 11 (3.2) 0 (0) 1.000

**Treated hypertension**, N (%) 127 (37.6) 7 (26.9) 0.399

***Diuretics***, N (%) 38 (11.2) 4 (15.4) 0.522

**Snoring**, N (%) 85 (25.1) 5 (19.2) 0.640

________________________________________________________________________________________________________________________

Abbreviations: **AST**, aspartate amino-transferase; **ALT**, alanine amino-transferase; **γ-GT**, gamma glutamyl transferase; **CPK**, creatine phosphokinase; **LDH**, lactate dehydrogenase; **HOMA-IR**, homeostasis model assessment of insulin resistance; **HbA1C**, glycosylated hemoglobin; **IGF-1**, insulin-like growth factor 1; **HDL**, high density lipoprotein; **LDL**, low density lipoprotein; **ApoB/ApoA-I ratio**, apolipoprotein A-I/ apolipoprotein B ratio; **SBP**, systolic blood pressure; **DBP**, diastolic blood pressure.

^a^ Data are reported as mean ( standard deviation) or counts ( percentages [calculated within single groups]).

^a^ By unpaired Student’s t test or Fisher’s exact test

**Supplementary Table 2.** Changes in anthropometric, clinical and metabolic features after the intervention (efficacy analysis; intention-to-treat population [N=364]) and according to gender.

__________________________________________________________________________________________________________________________________________________________________________________

**Females** (N=215) **Males** (N=149)

___________________________________________________ ___________________________________________________

**Baseline Day-14**  **Baseline Day-14**

**Characteristic** [Mean (SD)] [Mean (SD)]  **P-value** ^a^ [Mean (SD)] [Mean (SD)]  **P-value** ^a^ **P-value** ^b^

____________________________________________________________________________________________________________________________________________________________________________________

**Body weight**, kg 120.3 (18.3) 113.7 (18.2) <0.001 141.4 (26.4) 131.3 (25.1) <0.001 <0.001

**BMI**, kg/m^2^ 46.4 (6.4) 43.8 (6.4) <0.001 46.8 (8.3) 43.6 (8.1) <0.001 0.560

**Waist circumference**, cm 129.3 (14.6) 124.2 (15.0) <0.001 141.8 (16.1) 135.2 (15.2) <0.001 <0.001

**Hip circumference**, cm 139.7 (13.1) 135.9 (13.2) <0.001 137.8 (17.6) 133.1 (17.4) <0.001 0.271

**Waist-Hip ratio** 0.93 (0.08) 0.91 (0.08) <0.001 1.03 (0.06) 1.02 (0.06) <0.001 <0.001

**Uric acid**, mg/dL 5.1 (1.1) 7.4 (2.2) <0.001 6.4 (1.1) 8.6 (2.4) <0.001 <0.001

**Glucose**, mg/dL 95 (23) 80 (15) <0.001 102 (32) 81 (16) <0.001 0.031

**Insulin**, µU/mL 20 (15) 11 (11) <0.001 27 (24) 13 (13) <0.001 0.001

**HOMA-IR** 4.8 (4.2) 2.2 (3.0) <0.001 7.3 (7.8) 3.0 (3.7) <0.001 <0.001

**C-peptide**, ng/mL 3.9 (2.1) 2.3 (1.2) <0.001 4.8 (2.3) 2.8 (1.6) <0.001 <0.001

**HbA1C**, % 5.7 (0.8) 5.5 (0.7) <0.001 6.0 (1.2) 5.7 (1.0) <0.001 0.017

**Growth hormone**, ng/mL 0.82 (1.63) 1.90 (3.38) <0.001 0.30 (0.80) 1.28 (2.06) <0.001 <0.001

**IGF-1**, ng/mL 146 (81) 116 (72) <0.001 143 (67) 135 (81) <0.001 0.723

**Total cholesterol**, mg/dL 195 (34) 155 (37) <0.001 194 (35) 158 (38) <0.001 0.688

**HDL cholesterol**, mg/dL 50 (13) 38 (10) <0.001 41 (9) 33 (8) <0.001 <0.001

**LDL cholesterol**, mg/dL 124 (30) 96 (32) <0.001 127 (32) 103 (35) <0.001 0.308

**Triglycerides**, mg/dL 123 (65) 95 (34) <0.001 152 (84) 108 (42) <0.001 <0.001

**Triglycerides - HDL ratio** 2.8 (2.3) 2.7 (1.4) 0.498 4.0 (2.8) 3.6 (1.9) 0.023 <0.001

**ApoA-I**, mg/dL 148 (29) 114 (32) <0.001 134 (29) 107 (39) <0.001 <0.001

**ApoB**, mg/dL 95 (25) 86 (29) <0.001 99 (25) 94 (44) 0.033 0.216

**ApoB/ApoA-I ratio** 0.67 (0.23) 0.84 (0.61) <0.001 0.80 (0.52) 0.94 (0.46) <0.001 0.006

**SBP**, mmHg 131 (11) 124 (9) <0.001 138 (10) 129 (8) <0.001 <0.001

**DBP**, mmHg 79 (8.1) 74 (6.1) <0.001 84 (8.2) 77 (6.8) <0.001 <0.001

**Heart rate**, bpm 73 (3.6) 72 (3.6) 0.002 73 (3.7) 72 (2.5) 0.006 1.000

____________________________________________________________________________________________________________________________________________________________________________________

Abbreviations: **BMI**, body mass index; **HOMA-IR**, homeostasis model assessment of insulin resistance; **HbA1C**, glycosylated hemoglobin; **IGF-1**, insulin-like growth factor 1; **HDL**, high density lipoprotein; **LDL**, low density lipoprotein; **ApoA-I**; Apolipoprotein A-I; **ApoB**; Apolipoprotein B; **SBP**, systolic blood pressure; **DBP**, diastolic blood pressure.

^a^ Baseline vs. end of study by Student’s t-test for paired data

^b^ Between gender at baseline
